# Addressing the challenges of tuberculosis in pregnancy and postpartum: a question-and-answer-based review

**DOI:** 10.36416/1806-3756/e20250341

**Published:** 2026-11-09

**Authors:** Clara Sarquis Rodrigues, Lorrayne Isidoro Gonçalves, Clemax Couto Sant’Anna, Silvia S. Chiang, Anna Cristina C. Carvalho

**Affiliations:** 1. Laboratório de Inovações em Terapias, Ensino e Bioprodutos, Instituto Oswaldo Cruz, Fiocruz, Rio de Janeiro (RJ), Brasil.; 2. Faculdade de Medicina, Universidade Federal do Rio de Janeiro, Rio de Janeiro (RJ), Brasil.; 3. Department of Pediatrics, Alpert Medical School, Brown University, Providence, Rhode Island, USA.; 4. Center for International Health Research, Brown University Health, Providence, Rhode Island, USA.

**Keywords:** tuberculosis, pregnancy, neonate, prevention, treatment

## Abstract

In 2023, tuberculosis (TB) regained its position as the leading cause of death from a single infectious agent worldwide. Despite its global relevance, TB during pregnancy remains insufficiently understood, hindering the development of specific strategies for its diagnosis, treatment, and prevention in this population. Pregnant women with TB are at increased risk of maternal complications such as preterm birth, anemia, and postpartum hemorrhage, as well as higher mortality rates. Fetal complications include low birth weight, intrauterine growth restriction, and perinatal death. Transmission of TB from mother to child can occur via the transplacental route (resulting in congenital TB) or through close contact after birth (resulting in perinatal TB), especially when the mother has disseminated TB or smear-positive pulmonary TB, respectively. Early diagnosis is essential to minimize risks, interrupt transmission, and optimize clinical outcomes. For drug-sensitive TB, the standard treatment regimen is considered safe; however, treatment of drug-resistant TB requires the use of potentially teratogenic drugs. Prophylaxis with isoniazid and rifampicin has proven effective and safe in preventing TB in exposed neonates. The lack of clinical trials involving pregnant women with TB limits the available scientific evidence regarding the safest and most effective diagnostic and therapeutic approaches. This review, presented in a question-and-answer format, aims to contribute to improved management of TB during pregnancy and reduce the risk of adverse outcomes for both mother and child.

## INTRODUCTION

Until the onset of the COVID-19 pandemic, tuberculosis (TB) was the leading global cause of death from a single infectious agent.^1^ In 2023, TB once again occupied this position, with an estimated 10.8 million new cases reported that year.^2^ TB ranks among the top causes of morbidity and mortality worldwide, being considered the 10th leading cause of death in 2021.^3^ Globally, it is also one of the major causes of death among women of reproductive age and a significant non-obstetric cause of maternal mortality.^4^ ^,^ ^5^ Although there was a slight decrease in global deaths due to TB (1.25 million in 2023 vs. 1.32 million in 2022), the disease remains a major public health priority, particularly in settings with a high prevalence of HIV coinfection.^2^ Brazil is included on the World Health Organization (WHO) list of countries with a high TB burden, both in terms of total case numbers and TB-HIV coinfection rates.^6^

Despite efforts by national and international organizations to improve the quality of TB-related data, information on the disease in certain population groups-particularly pregnant and postpartum women (defined as up to 6 weeks after delivery)-remains scarce. Cohort studies have identified an increased risk of TB during pregnancy and the postpartum period,^5^ ^,^ ^7^ ^,^ ^8^ although the true burden of the disease at this stage of a woman’s life remains poorly understood. This lack of data hampers the accurate assessment of its magnitude and the development of appropriate diagnostic and therapeutic strategies for this population. Until recently, WHO documents made no mention of TB in pregnant women. However, in 2023, the organization published the “Roadmap towards ending TB in children and adolescents”,^9^ emphasizing that effective progress in TB control among children and adolescents requires targeted actions addressing TB in pregnant and postpartum women.

The WHO’s “Global Tuberculosis Report 2024”^2^ includes, in its annexes, global estimates from 2019 on TB and pregnancy, indicating that approximately 200,000 pregnant and postpartum women develop TB each year (151,000 during pregnancy and 49,000 in the postpartum), primarily in sub-Saharan Africa and Southeast Asia.^4^ The report also highlights major gaps in access to preventive therapies and adherence to TB treatment during pregnancy. It further notes that only 50% of pregnant and breastfeeding women undergo TB screening or receive preventive therapy in high-burden countries-factors that hinder appropriate clinical decision-making and risk mitigation for both mother and fetus.^2^ ^,^ ^4^

In Brazil, information on pregnancy status has been included in TB notification forms since 2007, enabling the monitoring of specific indicators for pregnant women. Tuberculosis during pregnancy accounted for 0.46% (n=390) of all new TB notifications in the country in 2023,^4^ but this figure is likely underestimated due to underdiagnosis or underreporting. Although TB screening during prenatal care is recommended in Brazil,^10^ the extent to which it is effectively implemented remains unclear. A recent study conducted in Brazil using data from the National Notifiable Diseases Information System (SINAN) from 2016 to 2022 found that, among 96,868 women with TB, 1,870 (1.9%) were pregnant. The study identified a significant association between pregnancy and unfavorable TB treatment outcomes, including a higher risk of death among pregnant adolescents and women aged 40-49 years.^7^ Limited knowledge about TB in pregnancy among healthcare professionals-leading to delayed diagnosis and treatment-may contribute to ongoing disease transmission during pregnancy, resulting in higher rates of spontaneous abortion and maternal mortality.^6^ ^,^ ^11^ ^,^ ^12^

During pregnancy, immune adaptations promote tolerance to the fetus but reduce defense mechanisms against *Mycobacterium tuberculosis*. The shift from a Th1 to a Th2 immune response decreases the production of cytokines, such as IFN-γ and TNF-α, which are critical for containing the infection. As a result, pregnant women who are more vulnerable may progress from latent to active TB infection.^13^ ^,^ ^14^ When active TB develops, chronic inflammation, maternal hypoxia, and nutritional stress can impair placental function and restrict oxygen and nutrient supply to the fetus. These mechanisms help explain why TB in pregnancy is associated with preterm birth, low birth weight, intrauterine growth restriction, and higher perinatal mortality.^15^ ^,^ ^16^ ^,^ ^17^

Significant gaps persist in the diagnosis and treatment of both active and latent TB in pregnant and postpartum women, such as underdiagnosis and suboptimal treatment, including congenital TB and the management of neonates exposed to *M. tuberculosis*. Addressing these gaps requires targeted interventions for these populations.^6^ ^,^ ^18^ This review, presented in a question-and-answer format, summarizes the most up-to-date concepts regarding TB management during pregnancy and the postpartum period. It covers maternal diagnosis and treatment, fetal risks, and preventive measures for newborns of mothers with pulmonary TB, with particular emphasis on the Brazilian Ministry of Health’s recommendations.

## METHODS

The present study is a narrative review^19^ on the clinical management of TB during pregnancy, conducted through the analysis of original articles and literature reviews published between January 2019 and January 2024. The consulted databases were PubMed and SciELO, and the keywords used in the search were “tuberculosis” and “pregnancy.” Articles in Portuguese, English, and Spanish were included. Based on this keyword search strategy, 132 studies were initially identified. After reviewing the titles and abstracts, 18 studies were selected, and an additional 25 manuscripts were identified through reference list screening, totaling 43 manuscripts. In addition, 12 additional documents (national and international TB manuals, including technical notes) were retrieved from the websites of the Brazilian Ministry of Health and the WHO and included in this review.

## QUESTIONS AND ANSWERS

### What is the clinical presentation of TB during pregnancy?

The clinical presentation of TB during pregnancy is similar to that observed in non-pregnant women.^15^ ^,^ ^20^ Common symptoms include persistent cough (with or without hemoptysis), fever, night sweats, fatigue, and weight loss. Dyspnea may occur as a result of diffuse involvement of the pulmonary parenchyma, as seen in miliary TB. However, diagnosing TB during pregnancy can be challenging because of the overlap between TB-related symptoms and physiological changes of pregnancy-such as fatigue and dyspnea-as well as weight gain, which can mask the clinical suspicion of the disease.^12^ ^,^ ^20^ ^-^ ^22^ As with pulmonary TB, extrapulmonary manifestations in pregnant women do not differ significantly from those observed in men or non-pregnant women.^21^

### How is active TB diagnosed in pregnant women?

The diagnosis of TB in pregnant women relies on a high index of clinical suspicion, a history of contact with individuals with pulmonary or laryngeal TB (the risk of disease progression is highest within the first two years after the initial infection, although infection acquired more than two years earlier does not exclude the disease), and the investigation of specific symptoms.^13^

For the diagnosis of active TB, the same diagnostic approach used for non-pregnant individuals is recommended. This includes microbiological methods such as sputum smear microscopy, rapid molecular testing (Xpert Ultra), and culture for *M. tuberculosis* with drug susceptibility testing, according to local TB program guidelines and resource availability.^23^ ^-^ ^25^ In some cases, procedures such as induced sputum collection, bronchoscopy (for patients unable to produce spontaneous sputum or those with endobronchial lesions), and biopsies (particularly for presumptive extrapulmonary TB) may be required to confirm the diagnosis.^13^

Chest radiographs during pregnancy should be performed with caution due to the potential risk of fetal radiation exposure. Radiographs must be obtained using lead abdominal shielding and, in specific cases, may be postponed until after the first trimester. However, it is important to emphasize that radiation exposure from chest radiographs is minimal, and the best available diagnostic tools for TB should be offered to pregnant patients.^10^ ^,^ ^23^

HIV serological testing is recommended for all patients with presumptive TB, as it enables the early initiation of antiretroviral therapy and reduces morbidity and mortality among coinfected individuals. Moreover, antiretroviral treatment during pregnancy decreases the risk of vertical HIV transmission.^25^ The WHO recommends TB screening in HIV-positive pregnant women based on four key symptoms: cough, fever, night sweats, and weight loss. However, this screening method has demonstrated low sensitivity in pregnant women, indicating that additional approaches-such as sputum testing, chest radiography, and even the tuberculin skin test (TST), when available-may be required for the early detection of TB in this population.^22^

In Brazil, TB screening is recommended for both low- and high-risk pregnant women during prenatal care, following the same diagnostic approach used for non-pregnant women and applying abdominal protection when performing chest radiography.^26^ ^,^ ^27^

### What are the risks of TB during pregnancy and childbirth?

The delayed diagnosis of TB during pregnancy, leading to disease progression, increases the risk of adverse outcomes for the mother, fetus, and newborn.^15^ ^,^ ^17^ ^,^ ^20^ ^-^ ^28^ ^,^ ^29^ In mothers, active TB is associated with a higher incidence of severe complications, including preeclampsia, eclampsia, chorioamnionitis, spontaneous abortion, preterm labor, postpartum hemorrhage, maternal anemia, and the need for cesarean delivery.^8^ ^,^ ^20^ ^,^ ^23^ ^,^ ^30^ Retrospective cohort studies conducted in the United States have identified anemia and postpartum hemorrhage as the most frequent complications among women with TB.^28^ ^,^ ^31^ ^,^ ^32^

Neonates born to mothers with TB are at increased risk of low birth weight, intrauterine growth restriction, neonatal mortality, birth asphyxia, and congenital TB.^7^ ^,^ ^30^ ^,^ ^16^ ^,^ ^33^ There is a 2-fold increase in the risk of preterm birth, low birth weight, and intrauterine growth restriction, as well as a 6-fold increase in perinatal mortality.^12^ ^,^ ^34^ Furthermore, HIV coinfection significantly increases the risk of both maternal and neonatal complications, raising maternal and infant mortality in regions with high HIV prevalence. In a study from South Africa, 54% of maternal deaths from TB were attributable to HIV-1 infection.^35^ ^,^ ^36^

Overall, the evidence suggests that untreated TB during pregnancy poses greater risks to both mother and child than the potential adverse effects associated with its treatment.^17^

### What is the risk of congenital and perinatal transmission of TB?

Congenital transmission of TB is rare but tends to be more frequent in pregnant women with miliary TB, tuberculous meningitis, tuberculous pleural effusion (which may indicate recent primary infection), pelvic or genital TB, or placental TB confirmed by histopathology.^7^ ^,^ ^24^ Transmission occurs via the transplacental route or through the umbilical vein, leading to the formation of a primary complex in the liver and subsequent secondary dissemination.^15^ ^,^ ^37^. Some authors also suggest that secondary transmission may take place through aspiration of infected amniotic fluid during delivery.^24^

The diagnostic criteria proposed by Cantwell et al. (1994) for congenital TB remain in use and include: (1) lesions present during the first week of life; (2) a primary hepatic complex or caseating hepatic granulomas; (3) TB of the placenta or maternal genital tract; and (4) exclusion of postnatal transmission, confirmed by the absence of contact with a mother with pulmonary TB or other infectious individuals, including healthcare workers potentially in close contact with the newborn. Reports have indicated that congenital disease may manifest later, up to three weeks after birth.^38^ Although rare, congenital TB is associated with high mortality-especially when symptoms appear early-and may present as sepsis with pulmonary involvement and hepatosplenomegaly.^38^

Perinatal transmission is considerably more common, with the highest risk observed in mothers with active pulmonary TB, particularly when smear-positive. Transmission may occur during delivery or through close contact with the mother after birth. The risk of postnatal transmission is influenced by factors such as maternal bacillary load, comorbidities (e.g., HIV infection), the effectiveness and duration of anti-TB therapy, and the proximity and duration of the newborn’s exposure to the mother or other infected individuals.^21^

### How is drug-sensitive TB treated in pregnant women?

The treatment of drug-sensitive TB during pregnancy follows the same regimen recommended for the general adult population and non-pregnant women and should be initiated as early as possible to reduce maternal and fetal morbidity and mortality. The standard regimen consists of a 2-month intensive phase with four drugs (isoniazid, rifampicin, ethambutol, and pyrazinamide), followed by a 4-month continuation phase with isoniazid and rifampicin.^18^ ^,^ ^25^ Although isoniazid and rifampicin cross the placenta, they are considered safe during pregnancy.^20^ These drugs are classified as Category C by the U.S. Food and Drug Administration (FDA), but no significant teratogenic effects have been observed to date.^39^ As for pyrazinamide, whether or not the drug crosses the placenta is not yet well established; however, despite its potential hepatotoxicity, widespread global use has demonstrated its safety in pregnancy.^40^ Ethambutol is also considered safe for use in this population.^1^

Strict monitoring of treatment adherence and adverse effects is essential to ensure efficacy and safety for both mother and fetus.^21^ The combination of isoniazid and pyrazinamide increases the risk of hepatotoxicity, and pregnant women should be counseled on early signs and symptoms of hepatitis. Liver function monitoring may be recommended.^13^ ^,^ ^25^ ^,^ ^40^ Administration of pyridoxine (vitamin B6) during pregnancy is advised to reduce the risk of isoniazid-associated neuropathy and to prevent seizures in exclusively breastfed infants whose mothers are receiving isoniazid treatment.^15^ ^,^ ^20^

Observational studies have shown that isoniazid administration in pregnant women with HIV is not associated with significant adverse pregnancy outcomes. Nevertheless, liver function monitoring remains crucial, especially in patients with preexisting liver disease or those receiving concomitant medications such as antiretroviral therapy.^23^ ^,^ ^41^ ^,^ ^42^

### How is drug-resistant TB treated in pregnant women?

The global impact of multidrug-resistant tuberculosis (MDR-TB-i.e., TB caused by *Mycobacterium tuberculosis* strains resistant to both rifampicin and isoniazid) in pregnant women remains poorly understood. Managing MDR-TB treatment in this population is particularly challenging due to the teratogenic potential of some second-line anti-TB drugs.^29^ However, favorable pregnancy outcomes can be achieved when pregnant patients are treated with effective regimens consisting of at least four active drugs, including pyrazinamide, fluoroquinolones, linezolid, and newer agents such as bedaquiline, delamanid, and pretomanid.^2^ ^,^ ^39^ ^,^ ^43^ Pregnant women receiving treatment with linezolid or terizidone are recommended to take pyridoxine supplementation.^25^

Table 1 lists the available anti-TB drugs and their respective safety profiles for use during pregnancy.

**Table 1:** Safety of anti-TB drugs for use during pregnancy.

Drug	Safety Category	Information
Para-aminosalicylic acid	C	Use with caution, only if essential. Not considered teratogenic.
Amikacin	D	Not recommended. Fetal developmental toxicity (e.g., congenital deafness).
Bedaquiline	B/C	Recent studies suggest safety in pregnant women in critical drug-resistant TB. Limited teratogenicity data.
Capreomycin	C	Possible fetal toxicity. Use after first trimester if essential; consider possible substitution.
Clarithromycin	C	Use with caution, if essential.
Clofazimine	C	Animal studies suggest teratogenic risk. Avoid during pregnancy.
Streptomycin	D	Avoid use. Fetal developmental toxicity (congenital deafness).
Ethambutol	B	Safety demonstrated in pregnant women.
Ethionamide	C	Contraindicated. Teratogenic effects observed in animals. Safer alternatives available.
Fluoroquinolones (Levofloxacin, Moxifloxacin)	C	Use with caution, if essential. Adverse effects observed in animal studies.
Isoniazid	C	Safety demonstrated in pregnant women. Use with pyridoxine (vitamin B6) during pregnancy.
Linezolid	C	Not recommended. Limited studies.
Pyrazinamide	C	Safety demonstrated in pregnant women.
Rifabutin	B	Not recommended.
Rifampicin	C	Safety demonstrated in pregnant women.
Rifapentine	C	Use only if benefits outweigh risks. Limited data.
Terizidone	C	Use with caution. Animal studies did not show fetal toxicity.
Delamanid	B/C	Recent studies suggest safety during 2nd and 3rd trimesters in critical drug-resistant TB.
Pretomanid	C	Limited data. Recommended only in MDR/XDR TB when benefits outweigh risks.
Cycloserine	C	Limited human data. Use with caution when essential. Safer alternatives available.
Clavulanic acid (with Amoxicillin)	B	Studies suggest safe use in pregnancy, especially for bacterial coinfections.
Imipenem/Cilastatin	C	Limited pregnancy data. Use only if no effective alternatives.
Tigecycline	D	Should be avoided during pregnancy due to fetal toxicity observed in some studies.

Source: Adapted from References 13, 25, 40, and 55. Safety classification for use in pregnancy: A- Established safety in human studies. B- Presumed safety based on animal studies. C- Uncertain safety; no human studies; some adverse effects seen in animals. D- Not recommended; evidence of risk in humans; use only if essential.

Infants born to women with MDR-TB or rifampicin-resistant TB (RR-TB) are at higher risk of *M. tuberculosis* infection, particularly during the first months of life, if the mothers receive inadequate treatment regimens.^24^ Maternal sputum smear positivity at the time of delivery and poor adherence to MDR/RR-TB treatment or antiretroviral therapy (ART) further increase the risk of mother-to-child transmission. These findings underscore the importance of supporting maternal treatment adherence and routine TB screening during prenatal and postpartum care, especially in high-incidence settings.^42^

## PREVENTION OF TB IN PREGNANT WOMEN AND NEWBORNS

### Is BCG vaccination recommended during pregnancy?

The Bacille Calmette-Guérin (BCG) vaccine is derived from live attenuated *Mycobacterium bovis* bacilli. Because it is a live attenuated vaccine, its use is not recommended during pregnancy due to the potential risk of disease caused by the attenuated bacillus. Although no fetal adverse effects have been reported in women who were inadvertently vaccinated during pregnancy, further studies are needed to confirm its safety.^44^ ^,^ ^45^

BCG vaccination is recommended at birth. However, in neonates born to mothers with active TB, vaccination may be postponed depending on the preventive treatment regimen indicated for the newborn (see the section below: “What is the appropriate management for a newborn of a mother with pulmonary TB?”).^44^ ^,^ ^45^

### How is latent TB diagnosed in pregnant women?

Latent TB infection (LTBI) occurs when an individual is infected with *M. tuberculosis*, but the bacillus remains viable without causing active disease at that time.^15^ The risk of progression from LTBI to active TB depends on several factors, primarily the host’s immune status.^6^ Diagnosis is based on positive immunological tests, such as the TST and interferon-gamma release assays (IGRAs), in the absence of clinical symptoms and chest radiographic abnormalities.^15^ ^,^ ^17^ ^,^ ^29^

The TST is considered safe during pregnancy; however, concerns have been raised regarding its sensitivity, which may be reduced due to the hormonal and immunological changes that occur during this period.^20^ ^,^ ^14^

### Should LTBI be treated during pregnancy?

LTBI treatment during pregnancy is recommended for women at high risk of progression to active TB. The most commonly used regimen is 6 to 9 months of isoniazid combined with pyridoxine supplementation. Treatment should ideally begin after the first trimester to minimize potential fetal risks.^21^ ^,^ ^17^ ^,^ ^46^ ^,^ ^47^ However, isoniazid use should be assessed individually due to the risk of hepatotoxicity.^48^ ^,^ ^49^

In addition to isoniazid, alternative LTBI treatment options include a 4-month rifampicin regimen or a 1- to 3-month once-weekly combination of rifapentine and isoniazid.^7^ ^,^ ^20^ ^,^ ^50^ While rifampicin is considered safe during pregnancy, safety data for rifapentine remain limited; therefore, its use during pregnancy is not recommended by the WHO.^49^

Some studies suggest postponing LTBI treatment until the postpartum period, unless the woman has a high risk of progression to active disease-such as recent contact with a patient with pulmonary or laryngeal TB or HIV coinfection. Similarly, Brazilian TB control guidelines similarly recommend deferring LTBI treatment until after childbirth, except in HIV-positive pregnant women, for whom preventive treatment is indicated after the first trimester.^25^

### What are the recommendations for delivery in pregnant women with TB?

The Brazilian Ministry of Health recommends that pregnant women with TB undergo monthly ultrasounds to assess fetal growth starting at 28 weeks of gestation, and that delivery occur no later than 40 weeks. Vaginal delivery is not contraindicated.^27^ If the patient is diagnosed with pulmonary TB at the time of delivery, appropriate respiratory precautions should be implemented to prevent aerosol transmission.

### Should breastfeeding be discontinued in mothers with pulmonary TB?

Breastfeeding should not be discouraged among mothers with TB, provided they are adherent to treatment.^6^ The use of a surgical mask during breastfeeding and infant care-especially while sputum smear microscopy is positive-is recommended to minimize the risk of transmission, without separating the newborn from the mother.^25^ Breast milk is not a recognized route of TB transmission, and the benefits of breastfeeding generally outweigh the potential risks associated with mother-to-infant transmission.^32^

Some evidence suggests that breastfeeding should be initiated after the mother has received at least two weeks of TB treatment. However, each case should be evaluated individually to ensure the newborn’s safety, particularly in mothers with multidrug-resistant TB, whose sputum may take longer to become negative.^20^ ^,^ ^27^

The presence of tuberculous mastitis contraindicates breastfeeding only from the affected breast.^27^

Information on the available anti-TB drugs and their use during breastfeeding is presented in Table 2.

**Table 2:** Safety of anti-TB drugs during breastfeeding.

Drug	Passage into breast milk	Recommendation during breastfeeding
Para-aminosalicylic acid (PAS)	Excreted into breast milk	Use with caution; monitor infant for gastrointestinal disturbances.
Amikacin	Low excretion into milk	Safe during breastfeeding; monitor infant’s hearing and renal function.
Bedaquiline	Excreted in small amounts	Not recommended due to limited data and potential cardiac toxicity.
Capreomycin	Limited data; likely minimal excretion	Use with caution; monitor infant’s hearing and renal function.
Clarithromycin	Excreted into breast milk	Use with caution; generally considered safe.
Clofazimine	Excreted into breast milk; may cause pigmentation	Use with caution; may cause skin discoloration in the infant.
Cycloserine	Excreted into breast milk	Use with caution; monitor infant for neurotoxic signs.
Delamanid	Limited data; probable excretion	Not recommended; lack of safety data.
Streptomycin	Excreted in small amounts	Safe during breastfeeding; monitor infant’s hearing.
Ethambutol	Excreted into breast milk	Safe during breastfeeding.
Ethionamide	Excreted into breast milk	Use with caution; may cause gastrointestinal effects; consider vitamin B6 supplementation.
Fluoroquinolones (Levofloxacin, Moxifloxacin)	Excreted into breast milk	Use with caution; generally well tolerated; monitor for GI effects.
Imipenem/Cilastatin	Excreted in small amounts	Use with caution; no significant adverse effects reported.
Isoniazid (standard doses)	Excreted into breast milk	Safe during breastfeeding; supplement vitamin B6 for mother and infant.
Isoniazid (high doses)	Excreted into breast milk	Use with caution; higher risk of hepatic toxicity; supplement vitamin B6.
Linezolid	Excreted into breast milk	Not recommended; risk of myelosuppression and hematologic toxicity.
Meropenem	Excreted in small amounts	Use with caution; generally considered safe.
Pyrazinamide	Excreted into breast milk	Safe during breastfeeding.
Pretomanid	Limited data; probable excretion	Not recommended; lack of safety data.
Rifabutin	Excreted into breast milk	Not recommended; limited safety data.
Rifampicin	Excreted into breast milk	Safe during breastfeeding.
Rifapentine	Limited data	Avoid use; rifampicin preferred.
Terizidone	Excreted into breast milk	Safe with monitoring; consider vitamin B6 supplementation.

Source: Adapted from References 25, 48, and 55.

### What is the appropriate management for a newborn of a mother with pulmonary TB?

Newborns of mothers with TB should undergo clinical evaluation for signs of active disease, which may include diagnostic tests such as chest radiography, gastric aspirate cultures, and cerebrospinal fluid examination.

In the absence of clinical or laboratory evidence of active TB, immediate initiation of window prophylaxis is recommended, with isoniazid being the most commonly used drug.^25^ ^,^ ^51^ In Brazil, the regimen has recently been updated to include rifampicin for 4 months as an alternative to isoniazid for 3 to 6 months, depending on the infant’s tuberculin skin test (TST) result.

Under both regimens, BCG vaccination should be deferred at birth. In Brazil, when the isoniazid regimen is used, the drug should be administered for 3 months, followed by a TST. If the test result is positive (≥ 5 mm induration), isoniazid should be continued to complete a 6-month course, and BCG vaccination should not be administered, as the child has already demonstrated an immune response to *M. tuberculosis*. If the TST is negative after 3 months, isoniazid should be discontinued, and BCG vaccination should be provided.^25^ In the rifampicin-based regimen, treatment lasts 4 months, no post-treatment TST is required, and BCG vaccination is recommended after prophylaxis is completed.^46^

Continuous clinical monitoring is essential for the early detection of active TB in exposed newborns.^51^ Both BCG vaccination and window prophylaxis are key interventions to reduce childhood TB morbidity and mortality.^2^ ^,^ ^6^ ^,^ ^13^ ^,^ ^25^ ^,^ ^52^ Additionally, pyridoxine supplementation is recommended for newborns receiving isoniazid prophylaxis and for those breastfed by mothers taking isoniazid, as the drug is excreted in breast milk.^53^ ^,^ ^54^

## FINAL CONSIDERATIONS

Given the magnitude of TB as a major cause of global morbidity and mortality, and the specific challenges posed by TB during pregnancy, the need for more targeted diagnostic and therapeutic strategies for this population is evident. Routine TB screening is recommended for pregnant women attending primary healthcare units and integrated prenatal and postpartum services in settings with a high disease burden. Screening through such programs may provide an effective approach for the early identification of active TB during pregnancy, thereby reducing the risk of adverse outcomes for both the mother and the newborn.

Current evidence remains limited, and uncertainties persist regarding the safest treatment modalities for pregnant women and their fetuses, particularly in patients with multidrug-resistant TB. These knowledge gaps largely reflect the exclusion of pregnant women from most TB clinical trials. Therefore, the development of safer and more effective therapeutic regimens during pregnancy, along with improved care strategies throughout the prenatal and postpartum periods, is essential to enhance TB-related outcomes, reduce adverse events, and minimize loss to follow-up among pregnant women and their children, ultimately contributing to better TB control in childhood.
